# Supplementary material for: Transplantation of Mesenchymal Stem Cells Derived from Old Rats Improves Healing and Biomechanical Properties of Vaginal Tissue Following Surgical Incision in Aged Rats
Source: Int J Mol Sci. 2024 May 24;25(11):5714. doi: 10.3390/ijms25115714 (PMC11172277; doi:10.3390/ijms25115714)
Supplement: Supplementary file 1 [file ijms-25-05714-s001.zip › ijms-2959149-supplementary.pdf]

Table S1: List of antibodies used in immunofluorescence staining, related to Materials and Methods.

Primary antibody	Species specificities	Secondary antibodies	Fluorochromes used	Catalog number	Vendor
Mouse anti rat CD68	Rat	Donkey anti-mouse	Alexa Fluor 488	MCA341R	BIO-RAD
Rabbit anti rat TNFa	Rat	Donkey anti-rabbit	Alexa Fluor 594	AAR33	BIO-RAD
Mouse monoclonal IgG CD31	Rat/Mouse/ Human	Donkey anti-mouse	Alexa Fluor 488/ Alexa Fluor 594	sc-376764	Santa Cruz Biotechnology
Rabbit polyclonal to MMP9	Mouse	Donkey anti-rabbit	Alexa Fluor 594	ab38898	Abcam
Mouse anti human muscle actin	Human, rat, mouse	Donkey anti-mouse	Alexa Fluor 594	M0635	Dako
